# Lack of Low Frequency Variants Masks Patterns of Non-Neutral Evolution following Domestication

**DOI:** 10.1371/journal.pone.0023041

**Published:** 2011-08-10

**Authors:** Céline H. Frère, Peter J. Prentis, Edward K. Gilding, Agnieszka M. Mudge, Alan Cruickshank, Ian D. Godwin

**Affiliations:** 1 School of Agriculture and Food Sciences, The University of Queensland, St Lucia, Queensland, Australia; 2 Biogeosciences Discipline, Queensland University of Technology, Brisbane, Queensland, Australia; 3 Agri-Science Queensland, Department of Employment, Economic Development and Innovation, Warwick, Queensland, Australia; Arizona State University, United States of America

## Abstract

Detecting artificial selection in the genome of domesticated species can not only shed light on human history but can also be beneficial to future breeding strategies. Evidence for selection has been documented in domesticated species including maize and rice, but few studies have to date detected signals of artificial selection in the *Sorghum bicolor* genome. Based on evidence that domesticated *S. bicolor* and its wild relatives show significant differences in endosperm structure and quality, we sequenced three candidate seed storage protein (kafirin) loci and three candidate starch biosynthesis loci to test whether these genes show non-neutral evolution resulting from the domestication process. We found strong evidence of non-neutral selection at the starch synthase IIa gene, while both starch branching enzyme I and the beta kafirin gene showed weaker evidence of non-neutral selection. We argue that the power to detect consistent signals of non-neutral selection in our dataset is confounded by the absence of low frequency variants at four of the six candidate genes. A future challenge in the detection of positive selection associated with domestication in sorghum is to develop models that can accommodate for skewed frequency spectrums.

## Introduction

Since the release of the seminal book ‘On the origin of species’ [1], there has been much interest in identifying the evolutionary processes underlying crop and animal domestication. Elucidating the origins of domesticated species can not only shed light on human history (e.g. [2], [3] but can also benefit future breeding strategies [4]. This is particularly evident when genes can be causally linked to adaptive phenotypes of interest in domesticated species (e.g. quality, pest resistance, drought tolerance, etc.).

Currently, two common approaches are used to find adaptive genes; the first is a top-down approach, and the second is the bottom-up approach [5]. Top-down approaches rely on QTL and LD mapping to isolate candidate genes associated with traits of interest and then use molecular population genetic methods to test for selection. In contrast, the bottom-up approach relies on molecular population genetic methods to identify regions or candidate genes under selection before aiming to link it back to a phenotypic trait. Population genetic theory predicts that intense directional selection (e.g. domestication) will lead to a significant loss of genetic diversity in the genomic region of selection (e.g. [6]). Thus, genes that have undergone selection during domestication should show a significant reduction in genetic diversity [7] and an excess of low frequency polymorphism [8]. Evidence for selection has been greatly documented in maize and rice (e.g. [9], [10], [11]), but few studies have to date detected signals of selection in *Sorghum bicolor* [12], [13].

*Sorghum bicolor* is a tropical grass that is considered the fifth most important cereal crop providing staple food for more than 500 million people worldwide. Domesticated sorghum has high levels of abiotic stress resistance, for example drought [14], heat [15] and salinity (e.g. [16], [17]) tolerance, making it the most important cereal crop in the semiarid zones of Sub-Saharan Africa. *Sorghum bicolor* was first domesticated in eastern Africa around 3000 to 6000 years ago [18], before being spread to regions including India (approx. 1500–1000 BC), the Middle East (approx. 900–700 BC) and the Far East (approx. AD 400).

The early stages of domestication through traditional farming methods likely focused on converting the small, shattering seeds of wild progenitors into the larger, non-shattering seeds we now see in domesticated cultivars and landraces of *S. bicolor* (Dillon et al 2007). While this modification would have led to significant changes in both the number of branches within the inflorescence and in the inter-node length of the rachis [19], the most significant phenotypic change occurred to the endosperm structure of domesticated cultivars (e.g. *S. bicolor*). Wild species are usually characterized by a single morphology across the entire endosperm, with varied protein bodies, starch granule size and shape [20], [21]. The endosperm of a cultivated *S. bicolor*, however, has two distinct regions or layers [20] called the floury central and corneous outer endosperm. Several studies have demonstrated that variation exists in the distribution and configuration of starch and protein between these two regions. This modification of endosperm structure affects the nutritional value of sorghum flours [22], [23], [24], [25]. Modern breeding programs, however, have focused on selecting for traits including photoperiod insensitivity, reduced height, drought tolerance and pest and disease resistance [26].

Based on the evidence that domesticated *S. bicolor* and wild relatives show significant differences in endosperm structure, we targeted three candidate seed storage protein (kafirin) loci and three candidate starch loci to test whether these genes show non-neutral evolution resulting from the domestication process. Identifying evolutionary bottlenecks is critical for the future of crop improvements as the maintenance of genetic diversity in crop species prevents genetic vulnerability and provides scope for future improvement [27], [28], [29]. Here, we present evidence of departures from neutral mutation-drift equilibrium and discuss the challenges associated with measuring positive selection in species characterized by low genetic diversity and complex demographic histories.

## Materials and Methods

### Plant Materials and DNA extraction

A total of 35 *S. bicolor* accessions were sampled for this study (Table 1). These 35 accessions were selected to include a wide range of phenotypic diversity in end-uses (breads, porridges, brewing, animal feed, broomcorns) and endosperm traits, which were measured using near-infrared reflectance spectroscopy. This germplasm was part of the Queensland Government Department of Employment, Economic Development and Innovation Diversified Gene Pool program [30]. DNA was extracted using a Qiagen DNeasy Plant Maxi Kit (QIAGEN®, Hilden, Germany).

**Table 1:** Accessions of *Sorghum bicolor* used in this study.

Sample Name	Country of Origin
B9401379-2-1-1-1-W	Australia
ETS 2174	Ethiopia
ICSV400	Mali
SC425-14E	Sudan
SPV 475	India
SC 49-14E	Sudan
SC 62-14E	Sudan
KARPER 669	USA
SC725-14E	Japan
BUDY	Kenya
TAM 422	USA
QL41	Australia
KS115	USA
BTx623	USA
R9188	USA
SC1017-14E	Ethiopia
SC1215-13E	Niger
SC424-14E	Japan
QL12	Australia
SU 2477	Sudan
“BLACK 430”	USA
SC382-14E	Nigeria
B9401379-2-1-1-1-N	Australia
Striker	Australia
SC798-14E	Sudan
A1*9_B004216/R002133	Australia
F9_R007620-2-1 b	Australia
BTx3054	USA
F4_B05049-2-4	Australia
F6_R04044-129	Australia
FF_B004214	Australia
IS 8525	Ethiopia
SC165-14E	Nigeria
SC265-14E	Burkina Faso
IS 25199	Ethiopia

### Targeted gene regions

To investigate the selective forces acting on starch biosynthesis and kafirin genes, we targeted three partial gene regions of starch metabolic pathway genes (branching, debranching and starch synthase genes) and three kafirin seed storage protein genes (β–, γ– and δ–kafirin genes). Using primer information from Hamblin et al. [31], we amplified a 1158 bp fragment of Branching Enzyme I (SBEI, Sb10g030776.1), a 1367 bp fragment of the Debranching Enzyme, pullulanase (PUL1, Sb06g001540.1), and a 1003 bp fragment of Starch Synthase IIa (SSIIa, Sb10g008200.1). Using primer information from Laidlaw et al. [32], we amplified a 785 bp fragment of the β -kafirin gene (bKaf, Sb09g000360.1), a 683 bp fragment of the δ–kafirin gene (dKaf, Sb10g013050.1), and a 813 bp fragment of the γ–kafirin gene (gKaf, Sb02g025510.1). Additionally, a 955 bp fragment of the *ADH1* gene (Sb01g008730.1) was amplified and used as neutral control locus according to the protocol of [33]. Primers and locations of landmarks within the amplicons for the gene models cited are given in Table S1. The PCR products were cleaned using ExoSAP-IT from Affymetrix Inc products (USA, usb.affymetrix.com). Cycle sequencing was conducted with the BigDye™ Terminator Cycle Sequencing Ready Reaction kit (Applied Biosystems, Foster City, California). After a MgSO_4_ clean-up, the sequencing fragments were run on an ABI 3730 DNA Sequencer (Applied Biosystems, Foster City, California). The sequences were visualised and edited manually using Geneious Pro version 5.0. The sequences have been submitted to GenBank (Submission number 1385804), see Table S2. See Table 1 for details about samples used in this study.

### Nucleotide diversity and neutrality tests

The software package DNAsp Version 4.20.2 (http://www.ub.es/dnasp) was used to calculate summary data statistics. We measured the number of polymorphic sites (*S*), the number of unique haplotypes (*h*), nucleotide diversity per-site (Pi (π)), [34], and Watterson's *Θ_W_* [35]. Median-joining networks [36] were constructed for each locus separately using polymorphic sites between haplotypes, both with and without indels as a fifth character state in Network program (Ver. 4.516, fluxus-engineering.com).

To detect departures of the site frequency spectrum from neutral expectations we used the following four summary statistics: Tajima's D [37]; Fay and Wu's *H* [38]; and Normalized Fay and Wu's H and normalized Zeng et al. ‘E test [39]. Each of these tests utilize the frequency spectrum distribution in different ways. Tajima's [37] D tests whether the frequency of low variants is higher or lower than high frequency variants. Fay and Wu's [38] H considers the abundance of high frequency relative to intermediate variants. Last, both the normalized Fay and Wu's H and the normalized E test allow for contrasting patterns between high and low frequency variants to be taken into account [39]. In order to control for background selection and demography, the significance of each test statistic described above was determined using DHEW compound test [40]. This test uses the combination of Tajima's *D*, Fay and Wu's normalized *H*, and the Ewens–Watterson test of neutrality to detect positive selection. The rejection region for this test was determined through 50,000 coalescent simulations conditional on the observed value of Θ_W_. We used a nominal threshold of *P* = 0.05 in the calculation of this region for each locus.

Finally, we used a polymorphism–divergence-based test, the Hudson–Kreitman–Aguade (HKA) test, to search for candidate genes with reduced levels of polymorphism relative to divergence. We used two outgroups for this test, a close relative (*S. propinquum*) and a distant relative (*Z. mays*). Two outgroups were chosen because of the high genetic similarity (99%) between *S. propinquum* and *S. bicolor* at many coding genes. This high level of sequence similarity may reduce the power of the HKA test to detect positive selection. *Zea mays* was therefore chosen as a second outgroup because it is more divergent and may increase the power of the HKQ test to detect positive selection. The HKA test [41] is one of the most commonly used [42] and has also been shown to be the most powerful test to measure positive selection with simulated data [43]. The HKA test compares patterns of polymorphism and divergence at multiple loci and uses coalescent parameters to estimate mutation rates based on the both the variation within species and divergence between species at a neutral loci. Significant departure from a constant ratio of polymorphisms to divergence among loci indicates a history of selection at the non-neutral gene; e.g. positive selection [43]. The HKA test was conducted using DNAsp Version 4.20.2. The ADH1 gene was used as a neutral control locus for the HKA test. As outgroups we used both a close (*S. propinquum*) and a distant (*Z. mays*) relative. Starch sequences from *S. propinquum* were kindly made available by Dr. Hamblin. Starch sequences from *Z. Mays* were downloaded from GenBank (SSIIa –AY499410.0, SBEI –AF072724.1, PUL1 –DQ195078.1). Kafirin sequences from *S. propinquum* and *Z. mays* were also downloaded from GenBank (*S. propinquum*: bKaf – GU732406.1, dKaf – GU732412.1, and gKaf – GU732410.1; *Z. mays* bKaf – EU952800.1, dKaf – EU952615.1, and gKaf – too divergent).

## Results

### Sequence Diversity

Measures of genetic diversity in the screened accessions (excluding outgroups) were generally high for cultivated sorghum, ranging from 0.72/kbp–7.93/kbp and 0.529/kbp–4.767/kbp for nucleotide diversity (π), and Watterson's *Θ_W_* respectively. Two starch biosynthesis genes, SBEI and SSIIa, and one kafirin gene, bKaf, displayed reduced nucleotide diversity compared to the other four genes sequenced (Table 2). The number of polymorphic sites within the kafirin loci varied from 3 to 11, while the number of haplotypes varied from 2 to 5 (Table 2). The number of polymorphic sites within the starch loci varied from 2 to 18, while the number of haplotypes varied from 3 to 6 (Table 2). Across our six candidate loci, we found only three nonsynonymous mutations: one at the bKaf locus and two at the PUL1 locus.

**Table 2:** Summary statistics of sequence variation at the six candidate loci.

Locus	Polymorphic Sites (S)	Haplotype (h)	Nucleotide Diversity (Pi)	*Θ*
SSIIa	5	4	0.00096±0.0002	1.338
SBEI	2	3	0.00072±0.0011	0.529
PUL1	18	6	0.00793±0.0013	4.767
bKaf	3	3	0.00160±0.0003	0.794
dKaf	8	2	0.00573±0.0020	2.142
gKaf	11	5	0.00481±0.0012	2.980

This table includes the number of polymorphic sites (S), the number of haplotypes (h), the nucleotide diversity per-site (Pi), and Watterson (Θ_W_).

### Haplotype Structure and Distribution

**Figure 1:**
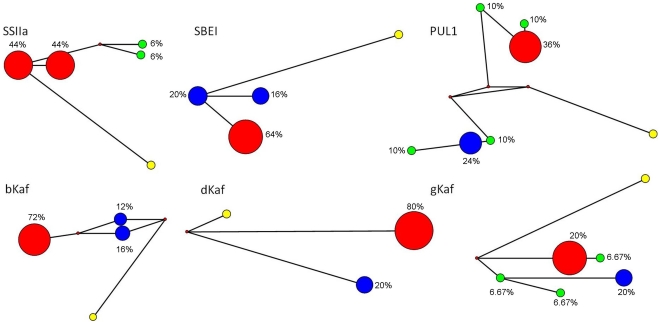
Median-joining networks. Red nodes represent high frequency haplotypes. Blue nodes represent intermediate frequency haplotypes. Green nodes represent low frequency haplotypes. And yellow nodes represent *Sorghum propinquum*.

The number of haplotypes within the starch loci varied from 3 to 6, while the number of haplotypes within the kafirin loci varied from 2 to 5 (Table 2). The frequency spectrum of different variants for all loci is best displayed in median-joining networks without indels (Figure 1). SSIIa displayed two high frequency haplotypes (44% each) and two low frequency haplotype (6% each). SBEI displayed one high (64%) and two intermediate (20% and 16%) frequency haplotypes. PUL1 displayed two groups of haplotypes. One group contained one high (36%) and two low (10% each) frequency haplotypes and the other contained one intermediate (24%) and two low (10% each) frequency haplotypes. bKaf displayed one high (72%) and two intermediate (16% and 12%) frequency haplotypes. dKaf displayed one high (80%) and one intermediate (20%) frequency haplotypes. Last, gKaf displayed one high (64%), one intermediate (16%) and three low (6.67%) frequency haplotypes.

### Tests of Neutral Evolution

We found strong evidence of non-neutral selection at the SSIIa gene with a significant Tajima's D and normalized E using the DHEW compound test (Table 3). The HKA test for the SSIIa gene showed a significantly decreased number of segregating sites than expected under neutral conditions with both outgroup species. The SBEI and the bKaf genes showed weaker evidence of non-neutral selection with significant Fay and Wu's H, normalized H using the DHEW compound test (Table 3). HKA tests for both loci also showed a significant reduction in the number of segregating sites expected under neutral evolution in the domesticated sorghum gene pool, but only with *Z. mays* as an outgroup. The PUL1 and dKaf genes showed evidence of non-equilibrium evolution using Fay and Wu's H and normalized H (Table 3). The other two loci gKaf and ADH1 displayed no evidence of either non-neutral or non-equilibrium evolution.

**Table 3:** Neutrality tests.

*S. propinquum*	*S. propinquum*	*Z. mays*
Locus	*D*	*H*	norm*H*	norm*E*	HKA (Obs/Exp SS)	
SSIIa	−0.72*	0.528	0.468	−0.913*	5/10.49*-	4/11.17*
SBEI	0.94	−0.146*	−0.237*	0.798	2/4.86	2/9.66*
PUL1	1.75	−0.900*	−0.324*	1.593	18/20.51	17/17.36
bKaf	0.39	−3.243*	−4.079*	3.639	3/6.07	4/13.7*
dKaf	0.26	−5.101*	−3.198*	3.052	8/8	9/13.8
gKaf	0.11	−0.758	−0.368	0.423	21/21.95	NA/NA

Significant departure from neutrality for the Tajima's *D*, Fay and Wu's *H*, norm*H* and norm*E* were assessed using the DHEW compound test. For the HKA test, we used ADH1 locus as the neutral locus and the following outgroups: *Sorghum propinquum* and *Zea mays*. The γ–kafirin gene showed little similarity between *Z. mays* and *S. bicolor*. As a result, the HKA was not conducted for this particular candidate locus (NA).

*indicates significant p-values.

## Discussion

The frequency of sequence polymorphism found in this study was consistent with previous studies in domesticated sorghum with one SNP every 82 bp, a value that is within the previously reported range of one SNP every 80–125 bp [13], [44]. In contrast, we observed a two-fold increase in the levels of sequence diversity (π) compared to those reported in previous sorghum studies [13], [44]. However, the level of sequence diversity at the three targeted starch loci was indistinguishable to the results for the same three genes in a recent study [31]. We deliberately selected a group of accessions with great diversity in endosperm traits and end uses and thus probably drove the high level of sequence diversity.

Consistent lines of evidence of positive selection associated with domestication have been demonstrated in a diverse range of crop species [10], [43], [45]. In maize, Whitt et al. [11] found evidence of positive selection at three starch pathway loci and Wright et al [46] estimated 2–4% of maize genome had been the target of recent artificial selection. Detecting similar evidence in sorghum has, however, been more problematic [13], [44]. In contrast to other studies that have examined evidence for positive selection at starch biosynthesis genes in sorghum, we found relatively consistent evidence of positive selection at the starch synthase IIa gene. In addition, we also found weaker evidence for patterns of non-neutral evolution at both the SBEI and the bKaf loci. Tajima's D was not significant at these two genes because of the lack of low frequency variants (Figure 1), a pattern previously observed for a number of genes in domesticated sorghum [44]. While both gKaf and PUL1 loci showed presence of low-frequency alleles, Tajima's D was found non-significant. This is because, both gKaf and PUL1 loci are also characterised by the presence of medium frequency variants, resulting in a non-significant Tajima's D (Figure 1). Overall our study further supports that the genome-wide excess of high-frequency alleles as well as the lack of low-frequency alleles in the *S. bicolor* genome may obscure signals of artificial selection. Indeed, Tajima's D relies on the site-frequency spectrum for detecting selection, and will therefore only be significant when a locus is characterised by the presence of low frequency and high frequency variants.

A confounding factor when detecting directional selection in tests with outgroups is the genetic divergence between the chosen outgroup and the target species at the locus of interest. While recognised as a powerful test to detect evidence of directional selection, the HKA test presented inconsistent results in this study. For example, we found evidence of directional selection at both SBEI and the bKaf loci when maize was the outgroup but not when *S. propinquum* was the outgroup. We speculate that the genetic similarity between *S. propinquum* and our samples of *S. bicolor* (99%) resulted in a lack of power to detect evidence of directional selection. Incorporating sequence information from multitude individuals of more distantly related wild sorghum species may help address this problem. The incorporation of multiple outgroup samples would be particularly important when conducting linkage disequilibrium analyses.

The difficulty in detecting selection at candidate gene loci in cultivated sorghum has been reported previously [44]. Ancestral population structure, a recent domestication bottleneck, subsequent expansions to new areas, founder effects in new areas and varieties, and introgression from wild relatives have all been suggested as possible complicating factors precluding the detection of selection in sorghum [13], [44]. The pattern of diversity to divergence and frequency spectrum of segregating sites in our study also confirm strong departures from a simple equilibrium model of evolution. The use of the DHEW compound test [40] helped in the detection of positive selection at candidate loci in this study. This compound test helps control for the confounding effects of complex demographic histories and population structure while detecting departures from neutral evolution at specific loci [47]. While, the identification of artificial selection in the *S. bicolor* genome has been difficult, we believe that the use of the DHEW compound test and more sophisticated future tests will allow more precise detection of artificial selection of crop genomes. These methodological advances combined with full genome resequencing of wild and domesticated sorghum genotypes will illuminate the effects of artificial selection on the *S. bicolor* genome.

## Supporting Information

Table S1 Primers and locations of landmarks within the amplicons for the gene models.
(DOC)

Table S2 Haplotype identity for each accessions of *Sorghum bicolor* used in this study for each gene referred to in GenBank (Submission number 1385804).
(DOC)
