# Supplementary material for: Lack of Low Frequency Variants Masks Patterns of Non-Neutral Evolution following Domestication
Source: PLoS One. 2011 Aug 10;6(8):e23041. doi: 10.1371/journal.pone.0023041 (PMC3154263; doi:10.1371/journal.pone.0023041)
Supplement: Table S1 — Primers and locations of landmarks within the amplicons for the gene models. (DOC) [file pone.0023041.s001.doc]

**Table S1|** Primers and locations of landmarks within the amplicons for the gene models.

| **Gene** | **Forward primer (5' to 3')** | **Reverse primer (5' to 3')** | **Total amplicon length (bp)** | **Sbi v1.4 gene model** |
| --- | --- | --- | --- | --- |
| *SSIIa* | TGGAGTCGATTTTGGATTTGTTTG | GGTCGTACAGCTCGAAATGTTG | 1003 | *Sb10g008200.1* |
|  | Features (base positions) | Intron 4 (1-61), Exon 5 (62-172), Intron 5 (173-272), Exon 6 (273-317), Intron 6 (318-593), Exon 7 (594-767), Intron 7 (768-927), Exon 8 (928-1003) | | |
| *SBEI* | CCAGAAAGGCTCCATTGAGGAA | GTGCCTGATCTGCTGCTAACC | 1158 | *Sb10g030776.1* |
|  | Features (base positions) | Exon 3 (1-53), Intron 3 (54-141), Exon 4 (142-211), Intron 4 (212-537), Exon 5 (538-804), Intron 5 (805-912), Exon 6 (913-1158) | | |
| *PUL1* | GTTGCGGAGTATTATCGCTTGG | AGGCTCAAAGGCTTCTAAAATCG | 1367 | *Sb06g001540.1* |
|  | Features (base positions) | Intron 1 (1-148), Exon 2 (149-166), Intron 2 (167-607), Exon 3 (608-743), Intron 3 (744-840), Exon 4 (841-893), Intron 4 (894-1012), Exon 5 (1013-1121), Intron 5 (1122-1202), Exon 6 (1203-1339), Intron 6 (1340-1367) | | |
| *bKaf* | GCTCCCACATATGCACTAGTCTATC | CCACACATGATAATGTGTGACGTC | 785 | *Sb09g000360.1* |
|  | Features (base positions) | Intergenic (1-42), 5'UTR (43-130), Exon 1 (131-709), 3'UTR (710-785) | | |
| *dKaf* | GCCAAGTCATCCAGCTTATCCAGC | GAGTCACATGCGATGGCATGTCAAC | 683 | *Sb10g013050.1* |
|  | Features (base positions) | Intergenic (1-180), Exon 1 (181-624), Intergenic (625-683) | | |
| *gKaf* | ACCCTCGTACGCCTATGC | CCATCGCCGCTGAATGAC | 813 | *Sb02g025510.1* |
|  | Features (base positions) | Intergenic (1-55), 5'UTR (56-100), Exon 1 (101-736), 3'UTR (737-813) | | |
| *ADH1* | AGGGCGGAAAGTTTGCGTCTTGAT | TGCCATCGCCAATCATCACACCCC | 955 | *Sb01g008730.1* |
|  | Features (base positions) | Intron 1 (1-65), Exon 2 (66-202), Intron 2 (203-299), Exon 3 (300-346), Intron 3 (347-785), Exon 4 (786-955) | | |
