# Supplementary material for: Lack of Low Frequency Variants Masks Patterns of Non-Neutral Evolution following Domestication
Source: PLoS One. 2011 Aug 10;6(8):e23041. doi: 10.1371/journal.pone.0023041 (PMC3154263; doi:10.1371/journal.pone.0023041)
Supplement: Table S2 — Haplotype identity for each accessions of Sorghum bicolor used in this study for each gene referred to in GenBank (Submission number 1385804). (DOC) [file pone.0023041.s002.doc]

**Table S2| Haplotype identity for each accessions of *Sorghum bicolor* used in this study for each gene referred to in GenBank (Submission number 1385804).**

|  | Haplotype Identity | | | | | | | |
| --- | --- | --- | --- | --- | --- | --- | --- | --- |
| Accessions | ADH1 | SBE1 | SSIIa | PUL1 | Beta-K | Delta-K | Gamma-K | |
| B9401379-2-1-1-1-W | 5 | 3 | 1 | 5 | 4 | 2 | 2 | |
| ETS 2174 | 5 | 3 | 2 | 5 | 4 | 2 | 2 | |
| ICSV400 | 5 | 2 | 2 | 5 | 1 | 2 | 2 | |
| SC425-14E | 5 | 3 | 2 | 1 | 4 | 2 | 2 | |
| SPV 475 | 4 | 3 | 2 | 2 | 1 | 2 | 2 | |
| SC 49-14E | 5 | 3 | 2 | 1 | 1 | 2 | 2 | |
| SC 62-14E | 5 | 1 | 2 | 1 | 4 | 1 | 2 | |
| KARPER 669 | 5 | 3 | 2 | 5 | 4 | 2 | 2 | |
| SC725-14E | 5 | 3 | 2 | 5 | 4 | 2 | 2 | |
| BUDY | 5 | 3 | 3 | 1 | 4 | 2 | 1 | |
| TAM 422 | 5 | 3 | 2 | 5 | 4 | 2 | 6 | |
| QL41 | 5 | 3 | 2 | 5 | 4 | 2 | 6 | |
| KS115 | 5 | 3 | 1 | 1 | 4 | 1 | 2 | |
| BTx623 | 5 | 3 | 2 | 5 | 4 | 2 | 6 | |
| R9188 | 5 | 3 | 2 | 5 | 4 | 2 | 6 | |
| SC1017-14E | 5 | 3 | 2 | 5 | 4 | 2 | 2 | |
| SC1215-13E | 5 | 2 | 2 | 1 | 4 | 1 | 7 | |
| SC424-14E | 5 | 3 | 2 | 5 | 4 | 2 | 2 | |
| QL12 | 4 | 3 | 2 | 3 | 3 | 1 | 2 | |
| SU 2477 | 5 | 3 | 1 | 5 | 4 | 2 | 2 | |
| “BLACK 430” | 5 | 2 | 1 | 4 | 4 | 2 | 2 | |
| SC382-14E | 5 | 3 | 1 | 1 | 3 | 2 | 2 | |
| B9401379-2-1-1-1-N | 2 | 1 | 2 | 1 | 4 | 2 | 2 | |
| Striker | 4 | 1 | 2 | 6 | 4 | 2 | 2 | |
| SC798-14E | 5 | 3 | 1 | 5 | 4 | 2 | 2 | |
| A1*9_B004216/R002133 | 5 | 3 | 1 | 5 | 4 | 2 | 2 | |
| F9_R007620-2-1 b | 4 | 2 | 2 | 5 | 4 | 2 | 6 | |
| BTx3054 | 4 | 3 | 2 | 1 | 4 | 2 | 3 | |
| F4_B05049-2-4 | 5 | 3 | 1 | 5 | 4 | 2 | 6 | |
| F6_R04044-129 | 1 | 3 | 2 | 1 | 4 | 2 | 6 | |
| FF_B004214 | 4 | 1 | 1 | 1 | 4 | 2 | 2 | |
| IS 8525 | 5 | 3 | 2 | 5 | 4 | 2 | 5 | |
| SC165-14E | 5 | 3 | 1 | 1 | 4 | 2 | 2 | |
| SC265-14E | 5 | 3 | 1 | 1 | 2 | 2 | 2 | |
| IS 25199 | 3 | 1 | 4 | 1 | 2 | 2 | 4 | |
|  | | | | | | | |  |
